# A randomised controlled trial of a personalised lifestyle coaching application in modifying periconceptional behaviours in women suffering from reproductive failures (iPLAN trial)

**DOI:** 10.1186/s12905-018-0689-7

**Published:** 2018-12-04

**Authors:** Ka Ying Bonnie Ng, Susan Wellstead, Ying Cheong, Nick Macklon

**Affiliations:** 1 Human Development and Health Academic Unit, Faculty of Medicine, University of Southampton, Southampton, SO16 6YD UK; 2 Department of Obstetrics and Gynaecology, Princess Anne Hospital, Level F, Coxford Road, Southampton, SO16 5YA UK; 3 Zealand University Hospital, University of Copenhagen, Copenhagen, Denmark; 4 London Women’s Clinic, 113-115 Harley Street, Marylebone, London, W1G 6AP UK

**Keywords:** Periconceptional health, Lifestyle, Online application, Smartphone application, Coaching, Randomised control trial

## Abstract

**Background:**

Lifestyle, in particular obesity and smoking has significant impacts on fertility and an important focus for the treatment of reproductive failures is the optimisation of periconceptional lifestyle behaviours. The preimplantation intrauterine environment within the uterus is also key for embryo development and early programming. Although the benefits a healthy periconceptional lifestyle are well described, there remains a paucity of data demonstrating the efficacy of interventions designed to optimise preconceptional lifestyle behaviours and choices.

**Methods:**

This study is a prospective randomised controlled trial which aims to address the question of whether an online personalised lifestyle coaching application is an effective means of delivering periconceptional advice in women suffering from reproductive failures. Women suffering from subfertility or recurrent miscarriages attending the outpatient clinic will be randomised into either the intervention arm (personalised online lifestyle coaching application) or the control arm (standard periconceptional advice including information from NHS websites). Both groups will be asked to complete a validated lifestyle questionnaire at baseline, and 6, 12, 18 and 24 weeks after randomisation. The primary outcome is the composite dietary and lifestyle risk score at 12 weeks. The secondary outcomes will include compliance with the program, proportion achieving spontaneous conception during the study period and the dietary and lifestyle risk score at 24 weeks.

**Discussion:**

With this study, we aim to clarify whether a personalised online based lifestyle coaching application is more effective at improving behaviours than standard advice offered by National Health Service (NHS) resources. A personalised lifestyle coaching application may represent an empowering and cost effective means of delivering periconceptional advice in women with subfertility or recurrent miscarriages.

**Trial registration:**

The iPLAN trial was retrospectively registered (ISRCTN 89523555).

**Electronic supplementary material:**

The online version of this article (10.1186/s12905-018-0689-7) contains supplementary material, which is available to authorized users.

## Background

The impact of lifestyle, and in particular obesity and smoking on fertility has been well described, and optimizing preconceptional lifestyle behaviours has become a primary strategy in the treatment of subfertility, recurrent pregnancy losses, and in particular the management of anovulation secondary to polycystic ovary syndrome [1]. It is also becoming clear that the preimplantation intrauterine environment is a key determinant of embryo development and early programming, and recent work has shown that the nutritional content of endometrial secretions is directly affected by diet [2].

The periconceptional period during which external and environmental factors exert significant influence on gamete and embryo health is considered to extend from 14 weeks pre-conception to 10 weeks gestation [3]. Factors shown to affect fertility and the chances of a healthy live birth include weight, diet, intake of vitamins, iodine and alcohol, smoking, substance abuse, environmental pollutants, infections, medical conditions, medications and family medical history [4]. Many important modifiable factors in the preconception period have also been shown to have important effects on the developing fetus [5, 6] with longer term impacts on the health of the offspring [7]. The detrimental effects of tobacco smoking have been well established [8, 9]; maternal tobacco smoking has been associated with subfertility, congenital malformations and deleterious effects on fetal and infant growth [10–12]. Nutrition is another modifiable factor; the occurrence of neural tube defects and altered fetal growth trajectories has been linked to deficiencies in folate, an essential vitamin B [13] and all woman planning a pregnancy are recommended 0.4 to 0.5 mg of folic acid per day for prevention of neural tube defects [14]. A ‘Mediterranean diet’ or a diet high in vegetable oils, fish and legumes and low in carbohydrate rich snacks in IVF patients have been shown to have positive effects on the level of red blood cell folate and vitamin B6 in the blood and follicular fluid, as well as increases the chances of pregnancy [15].

While there is a widespread recognition of the impact of preconceptional health on fertility outcomes and long term effects for the offspring, there is paucity of data demonstrating the efficacy of interventions designed to optimise preconceptional lifestyle behaviours and choices. One study has shown that tailored preconception counselling on lifestyle behaviours in subfertile couples in the outpatient tertiary clinic seems to reduce, in the short term, prevalence of harmful behaviours [16]. The NHS has a website which provides general information and advice for women planning a pregnancy [17]. For clinicians, there is general guidance offered by the National Institute of Health and care Excellence (NICE) on preconception advice and management [18]. The optimal method for delivering preconceptional lifestyle advice remains unclear. The use of personal or group lifestyle coaching can be costly and demanding, and there may be problems with non-compliance.

Recently, a novel online smartphone application delivering lifestyle coaching system has been developed, which may offer effective, low burden and low cost method of delivering peri-conceptional lifestyle advice. The ‘Smarter Pregnancy’ application addresses current priorities in the NHS; the NHS Five Year forward View, published in October 2014 argues for initiatives to empower patients and communities to take control of their health in order to help moderate rising demand on the NHS [19]. This online smartphone application may enable woman to be more in control of their health before and during pregnancy and may assist them in making lifestyle choices.

Whilst this online lifestyle application has been piloted, studies have only focussed on women in the primary care setting (University College London group [20]) and women and men who are generally seeking to become pregnant within 6 months (Rotterdam Group [21]). The effectiveness of the smartphone application in changing lifestyle choices has not been studied specifically in women with subfertility or in those suffering from recurrent miscarriages. Providing advice about factors affecting fertility and pregnancy outcomes in these women is a crucial step in helping them make lifestyle modifications to increase their chances of timely conception and chances of delivering a healthy, live baby.

## Methods and design

### Study objective

The prospective randomised controlled trial (the iPLAN trial ‘Impact of a Personalised Lifestyle coaching phone ApplicatioN in modifying peri-conceptional behaviours) aims to address the question of whether an online based lifestyle coaching application is an effective means of delivering periconceptional advice in women suffering from reproductive failures.

### Hypothesis

The hypothesis being tested in this study is that a smartphone based online lifestyle coaching application will be a more effective means of modulating periconceptional lifestyle behaviours compared to conventional measures of periconceptional counselling through standard information provided by NHS websites and patient information leaflets.

### Study population and recruitment

All women who attend the outpatient department in Princess Anne Hospital, Southampton and Salisbury District Hospital who meet the inclusion and exclusion criteria will be invited to participate in the iPLAN study. Women participating in the iPLAN trial will be women suffering from subfertility or recurrent miscarriages, aged between 18 and 45 years and actively trying to conceive. They will also need to be fluent in the use and understanding of English, and have a smartphone capable of running the online application.

Exclusion criteria include women who are on a specific diet for medical reasons, women with insulin diabetes and those undergoing any other means of lifestyle coaching, for example personal trainer or group lifestyle coaching.

Eligible patients will be informed about the iPLAN study during their first outpatient appointment and consultation with the medical team. In addition, patients can self-refer by contacting the research team, the details of which are on recruitment posters displayed in the outpatient departments of the participating hospitals. Eligible patients who wish to participate will need to provide written consent. After this, the patient will be given a unique activation code to the online lifestyle coaching application. Once registered, participants will be randomised by the application.

### Study design

**Fig. 1:**
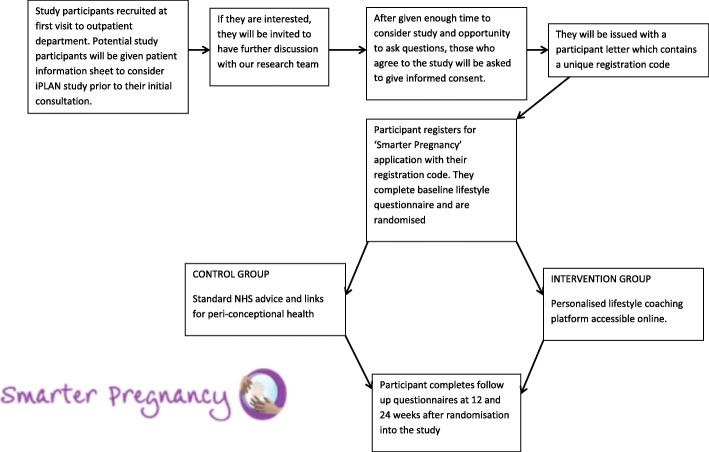
Flow diagram for the iPLAN study design

This study is a two-centre randomised control trial of using an online smartphone application in providing lifestyle coaching and modifying lifestyle parameters in women attending the outpatient department in Princess Anne Hospital, Southampton and Salisbury District Hospital. A flow diagram of the study design is shown in Fig. 1. Participants seen in the outpatient clinic suffering from infertility or recurrent miscarriages, who may be suitable for the study will be referred by the clinician to the research nurse. The research nurse will explain in detail about the study, ensure that the inclusion and exclusion criteria are met and takes informed written consent.

Women from both intervention and control group will be asked to visit the ‘Smarter Pregnancy’ website (www.smarterpregnancy.org.uk) and will be asked to register and activate their account by a unique validation code. Women will be asked to complete a baseline lifestyle questionnaire which assesses parameters including smoking habits, alcohol consumption, diet, exercise and weight, and then will be randomised by the ‘Smarter Pregnancy’ computer programme to either intervention arm or control arm. Both intervention and control group login to ‘Smarter Pregnancy’ using their personalised credentials created at registration.

In the intervention arm, women will have access to a personalised smartphone lifestyle coaching program. Through baseline and follow up lifestyle questionnaires (at 6, 12, 18 and 24 weeks) sent out via email, tailored lifestyle advice is generated; emails with tips, facts and recipes are sent to the intervention arm participants to encourage them to change unhealthy habits and maintain healthy habits. Coaching is focussed on study subjects who report inadequate intake of vegetables and fruit, and absence of folic acid supplementation, and those with unfavourable alcohol and smoking habits, which are identified by the baseline and follow up questionnaires. The smartphone application allows the women to update their pregnancy status; if the subject is pregnant, lifestyle advice is adapted for the pregnancy. Those randomised to the control arm will have access to standard periconceptional advice, including information from NHS websites.

The results will be analysed to determine whether a smartphone lifestyle coaching application can improve lifestyle behaviours in the periconceptional period, through a validated lifestyle questionnaire (see Appendix 1) at 6, 12, 18 and 24 weeks after randomisation. Patient compliance with the system at specific time points (at 6, 12, 18 and 24 weeks after randomisation) and the proportion of women achieving spontaneous conception during the study period will also be assessed. The expected length of time each subject will participate in the study for is 6 months after randomisation.

### Primary and secondary endpoints

The primary endpoint of the study is the composite dietary and lifestyle risk score at 12 weeks after randomisation. Secondary endpoints include (i) percentage of patients remaining compliant with system at 12, 18 and 24 weeks after randomisation, (ii) proportion achieving spontaneous conception during study period and (iii) composite dietary and lifestyle risk score at 24 weeks after randomisation.

### Participating hospitals

This is a two centre study that will be conducted in the outpatient departments of the Princess Anne Hospital, Southampton and Salisbury District Hospital. These are part of the University Hospital Southampton NHS Foundation Trust and Salisbury NHS Foundation Trust respectively.

### Randomisation

Participating women will be randomised in program by the computer generation of a series of validation codes which is unique for each participant. After completion of the baseline questionnaires, women will be randomised to the intervention or the control group. The randomisation process will be concealed and the research team will be blinded to the randomisation.

### Data collection

All women giving informed consent will be asked to complete an electronic registration and baseline questionnaire using their computer, laptop, tablet or smartphone. The validated lifestyle questionnaire is done at baseline, at 6, 12, 18 and 24 weeks after randomisation. Subjects will be sent an email with a link to the questionnaire at the time points specified. The lifestyle questionnaire includes assessment of folate and vitamin D intake, pregnancy status, BMI, diet (including fruit, vegetables, meat and/or meat substitutes, liver and/or liver products, shellfish and fish products, savoury snacks, sweet snacks, bread and rice, and ready-made meals and fast food), smoking status, alcohol intake and exercise. The full questionnaire is detailed in ‘Additional file 1’.

### Statistical analysis

#### Sample size calculation and power considerations

In order to show a difference in the proportion of participants achieving a high composite lifestyle score from 30% in the control arm to 50% in the study arm after 24 weeks of the intervention, with 80% power at a *P* value of < 0.05, 93 patients will be required in each arm. We have assumed a randomisation rate of 50% and assuming a drop-out rate of 15–20%, 220 patients will be randomized to each arm (440 patients to be recruited in total).

#### Data analysis

The difference between two groups for the composite score will be expressed as a proportion of those achieving high scores at the end of the intervention period. Chi squared testing will be applied to test these categorical variables for significance. Differences in parameters will be tested by a two-tail Mann Whitney U test. In order to adjust for predefined confounders such as age, socioeconomic class, educational attainment, working status and ethnicity, multiple regression analysis will be performed.

Missing data will be described, for example, by presenting the number and percentage of individuals in the missing category. All data collected on collection forms will be used, since only essential data items will be collected. No data will be considered spurious in the analysis since all data will be checked and cleaned before analysis. Range checks, identification of extreme values (Mean +/− 3*Std Dev) and consistency checks will be used to identify possible data errors.

An intention to treat analysis will be performed. No interim analysis is planned.

## Discussion

With this study, we aim to clarify whether a personalised online smartphone-based lifestyle coaching application is more effective at improving behaviours than standard advice offered by NHS resources. In addition, we aim to assess compliance with the application at 6 week timepoints and assess rates on spontaneous conception. A personalised lifestyle coaching application may represent an empowering and cost effective means of delivering periconceptional advice in women with subfertility or recurrent miscarriages.

## Additional file

Additional file 1: Full baseline lifestyle questionnaire. (DOCX 14 kb)

## Abbreviations

GCP: Good Clinical Practice
iPLAN: Impact of a Personalised Lifestyle coaching phone ApplicatioN in modifying peri-conceptional behaviours
IRAS: Integrated Research Application System
NHS: National Health Service
NICE: The National Institute of Health and care Excellence
