# Supplementary material for: A randomised controlled trial of a personalised lifestyle coaching application in modifying periconceptional behaviours in women suffering from reproductive failures (iPLAN trial)
Source: BMC Womens Health. 2018 Dec 4;18:196. doi: 10.1186/s12905-018-0689-7 (PMC6280350; doi:10.1186/s12905-018-0689-7)
Supplement: Supplementary file 1 — Full baseline lifestyle questionnaire. (DOCX 14 kb) [file 12905_2018_689_MOESM1_ESM.docx]

**Additional file 1:** Validated lifestyle questionnaire

Folate

1. Are you taking folic acid?

Vitamin D

1. Are you taking vitamin D as a separate tablet or just take multivitamins containing vitamin D?

Pregnancy

1. Are you pregnant?

BMI

1. How tall are you in cm?
2. What is your height in Kg?
3. Are you actively trying to lose weight now?

Vegetables

1. Last week, HOW MANY DAYS did you eat cooked vegetables (boiled/stir fried or cooked another way)?
2. On the days you ate cooked vegetables, HOW MANY PORTIONS did you have per DAY?
3. Last week, HOW MANY DAYS did you eat raw vegetables (like lettuce, tomatoes cucumber)?
4. On the days you ate raw vegetables, HOW MANY PORTIONS did you have per DAY?
5. Are you planning to eat DAILY a minimum of 2 PORTIONS of vegetables?

Fruit

1. Last week, HOW MANY DAYS did you drink a healthy fruit juice (for example, orange juice, freshly juiced or from a bottle WITHOUT any added sugars)?
2. On the days you drank fruit juice, HOW MANY GLASSES did you drink DAILY (one glass equal to 250ml or half a pint)?
3. Last week, HOW MANY DAYS did you eat apples, pears, bananas, oranges or other citrus fruits?
4. On the days that apples, pears, bananas, oranges, HOW MANY did you eat per DAY?
5. Last week, on HOW MANY DAYS did you eat any other fruit (including canned, tinned or dried fruit)?
6. Are you planning to eat a minimum of 2 portions of fruit DAILY?

Meat and/or meat substitutes. E.g. chicken, pork, beef, venison, goat, mutton, tofu and soya products

1. Last week, HOW MANY DAYS did you eat meat and/or products containing meat (including chicken, pork, beef, venison, lamb, quorn, tofu and soya meat alternatives- NOT soya milk or soya yoghurt)?
2. How many portions per day did you eat?

Liver and/or liver products. E.g. pigs liver, chicken liver, foie gras, liver sausage

1. Last week, HOW MANY DAYS did you eat liver and/or products containing liver?

Fish, shellfish and fish products. E.g all kinds of raw, cooked, fried fish, shrimp, lobster etc, fish finger, fish nuggets

1. Last week, HOW MANY DAYS did you eat seafood, fish or products containing fish?

Savoury snacks. E.g. fries, croquettes, potato chips, fried rice, hot dog, sausage rolls, spring rolls

1. On how many days last week did you eat savoury snacks? (including crisps, salted nuts, dried soups and sauces)

Sweet snacks. E.g. slice of cake, pastry, cookies, sweets

1. On how many days last week did you eat sweet snacks? (including cakes, biscuits, sweets and chocolate etc.)

Bread and rice. E.g. white breads, different types of bread, white rice species, other types of rice

1. Last week, HOW MANY DAYS did you eat BREAD?
2. On HOW MANY DAYS did you eat WHITE bread?
3. Last week, HOW MANY DAYS did you eat rice or pasta?
4. On how many days did you eat WHITE rice or pasta?

Ready-made meals and fast food. E.g. pizza, Chinese, kebab, hamburgers

1. On how many days in the last week did you eat ready meals and fast food? (such as takeaway food, kebab, fish and chips and Chinese, Indian etc.)

Smoking

1. Last week, HOW MANY CIGARETTES did you smoke?

Alcohol

1. Last week, HOW MANY alcoholic DRINKS did you drink?

Exercise

1. Over the past week, HOW MANY HOURS did you spend doing light exercise (household tasks, light physical work, walking)?
2. Over the past week, HOW MANY HOURS did you spend doing moderate exercise (walking fast, riding a bike, gardening)?
